# Comparison of the Transcriptome Response within the Swine Tracheobronchial Lymphnode Following Infection with PRRSV, PCV-2 or IAV-S

**DOI:** 10.3390/pathogens9020099

**Published:** 2020-02-05

**Authors:** Laura C. Miller, Damarius S. Fleming, Kelly M. Lager

**Affiliations:** 1 Virus and Prion Research Unit, National Animal Disease Center, USDA, Agricultural Research Service, Ames, IA 50161, USA; damarius.fleming@ars.usda.gov (D.S.F.); kelly.lager@ars.usda.gov (K.M.L.); 2 Oak Ridge Associated Universities/Oak Ridge Institute for Science and Education, Oakridge, TN 37830, USA

**Keywords:** PRRSV, PCV-2, IAV-S, lymph node, immune response, differential expression

## Abstract

Porcine reproductive and respiratory syndrome virus (PRRSV) is a major respiratory pathogen of swine that has become extremely costly to the swine industry worldwide, often causing losses in production and animal life due to their ease of spread. However, the intracellular changes that occur in pigs following viral respiratory infections are still scantily understood for PRRSV, as well as other viral respiratory infections. The aim of this study was to acquire a better understanding of the PRRS disease by comparing gene expression changes that occur in tracheobronchial lymph nodes (TBLN) of pigs infected with either porcine reproductive and respiratory syndrome virus (PRRSV), porcine circovirus type 2 (PCV-2), or swine influenza A virus (IAV-S) infections. The study identified and compared gene expression changes in the TBLN of 80 pigs following infection by PRRSV, PCV-2, IAV-S, or sham inoculation. Total RNA was pooled for each group and time-point (1, 3, 6, and 14 dpi) to make 16 libraries—analyses are by Digital Gene Expression Tag Profiling (DGETP). The data underwent standard filtering to generate a list of sequence tag raw counts that were then analyzed using multidimensional and differential expression statistical tests. The results showed that PRRSV, IAV-S and PCV-2 viral infections followed a clinical course in the pigs typical of experimental infection of young pigs with these viruses. Gene expression results echoed this course, as well as uncovered genes related to intersecting and unique host immune responses to the three viruses. By testing and observing the host response to other respiratory viruses, our study has elucidated similarities and differences that can assist in the development of vaccines and therapeutics that shorten or prevent a chronic PRRSV infection.

## 1. Introduction

Respiratory diseases are extremely costly to the swine industry worldwide and ongoing research is essential to gain a better understanding of the pathogenesis, diagnosis, and prevention of respiratory disease [1,2,3,4]. The intracellular changes that occur following infection by a virus are, for the most part, poorly understood. It is known that viruses hijack the biosynthetic, metabolic and signaling machinery of the cell for their own ends. Viral proteins interact with specific cellular components to alter the function of these pathways and even alter gene expressions in the host cell to bring about the successful replication and production of the progeny virus [5,6,7]. The cell has a number of innate mechanisms to detect the diversion of these functions and will initiate events to inhibit viral replication or to kill itself in an attempt to stop the infection [8,9,10,11]. These events, and how effective they are, have a profound effect on the events that follow. These include the ability to respond to and end the infection at the cellular or organismal level and whether pathological changes occur that may, in severe cases, lead to death. A major target of the biological analysis is to establish a relationship between the messenger RNAs that are transcribed from the genome and the regions that control their expression, the promoters that decipher which gene expression networks to regulate, and the transcription factors that act as master regulators of transcriptional control. One of the key immune organs involved in respiratory diseases are the tracheobronchial lymph nodes (TBLN). The lymph node is the place where the innate (early, non-specific) immune response talks to the adaptive (later, specific) immune system with the TBLN working specifically to drain lymphatic fluids from virus-infected lung tissues. While the TBLN contains a number of cell types, the advantage of sampling the TBLN is that both directly effect the virus on cells in the lymph nodes, as well as indirect effects on lymph nodes draining the lungs can be examined, giving our study an indication of the real host response [12,13]. While PRRSV, PCV-2, and IAV-S can induce respiratory disease in pigs that may appear somewhat clinically similar, [13,14,15,16], the cellular specificities, kinetics of clinical disease onset and duration of infection are different for each virus. PRRSV and PCV-2 can directly affect cells within lymph nodes as well as cause inflammation in the lungs [17,18,19]. 

The overall aim of this study is to acquire a better understanding of porcine respiratory disease by comparing gene expression changes within the porcine TBLN during the first two weeks of respiratory viral infections. Part of this project was dedicated to carrying out the analysis using previously collected sequence data to make the best use of our resources. The objective was to apply new bioinformatics techniques to previously collected and unused sequence data. The data for the virally infected TBLNs was originally sequenced using an older tag-based sequencing method similar to what is known as SAGE (Serial Analysis of Gene Expression) sequencing [20], referred to as Digital Gene Expression Tag Profiling (DGETP). Considered to be an improvement, DGETP was the most advanced derivate of SAGE for the analysis of expressed genes in eukaryotic organisms at the time of data collection. With DGETP, a specific segment from each transcribed gene recovers from the tissue under study, sequenced and counted, thus providing a transcription profile revealing what genes are transcribed and how often [21,22,23]. Despite the age of the samples and sequencing methods, the intersection in host response between these viral infections is still of interest as the results give some indication of how the host response has changed, if at all, over time, and gives a way to compare, contrast, and characterize genes and regulatory pathways that share the immune response to these major porcine infections.

By applying new computational methods to previous generation DGTEP sequencing results, our study identified genes that showed statistically significant changes in mRNA expression and intersected between pathogens during experimental infection in vivo. Understanding the host response by studying gene expression across multiple respiratory diseases may help to uncover biological functions that intersect between conditions. The information gleaned from this perspective has the potential to unlock new viewpoints for analysis—information that could prove key to many vaccinate-to-eradicate programs. 

## 2. Results

### 2.1. Clinical Evaluation and Gross Pathology

**Figure 1:**
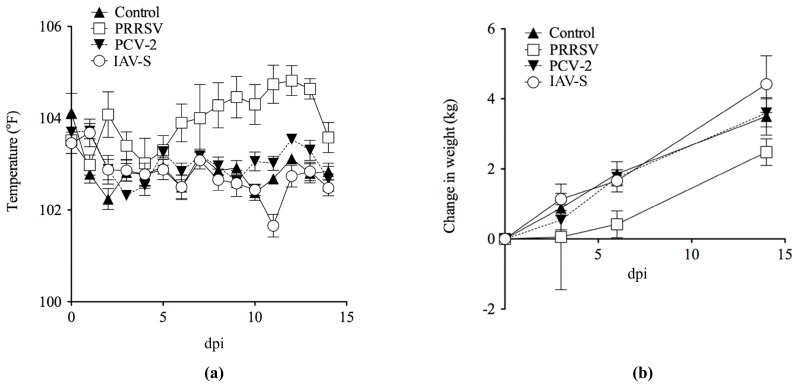
(**a**) Rectal temperature of pigs before and after challenge. Shown are mean rectal temperatures and standard error bars for each of the four groups. Pigs inoculated with porcine reproductive and respiratory syndrome virus (PRRSV) had a biphasic increase in rectal temperature with an initial peak at 2 dpi and a second sustained increase between 6 to 14 dpi. Pigs inoculated with porcine circovirus type 2 (PCV-2) had only a slightly increased rectal temperature from 10 to 14 dpi. Pigs inoculated with influenza A virus (IAV-S) had a transiently increased rectal temperature from 1 to 2 dpi; (**b**) body weight of pigs before and after challenge. Shown are mean body weights and standard error bars for each of the four groups.

The PRRSV, IAV-S and PCV-2 viral infections followed a clinical course in these domestic pigs typical of experimental infection of young pigs with these viruses [24,25,26]. PRRSV isolate SDSU-73 was the most pathogenic virus over the 14 day study, inducing a biphasic febrile response with an initial peak at 2 dpi (Figure 1a), followed by a sustained febrile response 6–14 dpi along with anorexia, lethargy, and dyspnea. PCV-2-infected pigs had only a very mild febrile response from 10–14 dpi, while the IAV-S group had a mild febrile response from 1–2 dpi (Figure 1a). Weight gain (Figure 1b) was the highest in pigs inoculated with IAV-S (0.31 kg/day), followed in decreasing order by pigs inoculated with PCV-2 (0.26 kg/day), control pigs (0.25 kg/day), and PRRSV (0.18 kg/day). The PRRSV-inoculated pigs had lungs with diffuse tan mottling at 14 dpi. The IAV-S-inoculated pigs had interstitial edema and dark areas on the lung surface at 3 dpi. PCV-2-inoculated pigs and control pigs had negligible macroscopic lesions. No significant bacteria were isolated from the BALF of any of the pigs. Macroscopic lung lesion scoring (Table 1) paralleled the disease severity with PRRSV, having maximal lung involvement (57%) at 14 dpi, IAV-S maximal at 6 dpi (31%) and PCV-2 only involving 1% of the lungs. While PRRSV, PCV-2, and IAV-S can induce respiratory disease in pigs that may appear similar depending on the time-course of infection, the mechanism for each virus is different, reflecting their unique properties. PCV-2 and PRRSV can directly infect cells within lymph nodes, as well as cause inflammation in lungs. IAV-S does not typically directly infect cells within lymph nodes, but the lymph node cells will be affected by the contents of the lymphatic fluids drained from the inflamed pneumonic tissues. 

#### 2.1.1. Serological Analysis

Only pigs inoculated with IAV-S seroconverted to this virus at 14 dpi; pigs in the rest of the groups remained seronegative to IAV-S during the experiment. The PRRSV-inoculated pigs seroconverted at 14 dpi. Antibodies to PRRSV were only detected in PRRSV-inoculated animals; animals in the rest of the groups remained seronegative to PRRSV during the entire experiment. The PCV-2 maternal antibody status was assessed prior to the animal study and the antibodies to PCV-2 in all groups were detected at a low level during the entire experiment. 

#### 2.1.2. Quantitative PCR for Virus Nucleic Acid and Virus Isolation

Table 2 summarizes the virus detection assays completed. In this study the IAV-S used is from the same genetic cluster as the 2009 novel A/H1N1. The IAV-S-inoculated pigs were no longer shedding the virus at 14 dpi and all the sera tested were negative by real-time RT-PCR and virus isolation at all time points tested.

### 2.2. Differentially Expressed Gene (DEG) Analysis During Infection

**Figure 2:**
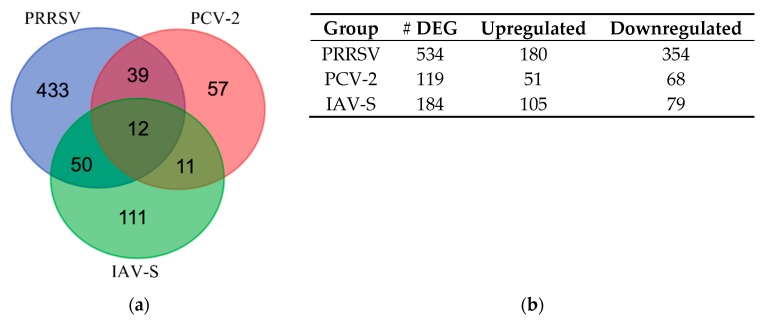
Venn diagram intersection of PRRSV, PCV-2, and IAV-S. (**a**) Intersection of Differentially Expressed Gene (DEG) in response to PCV-2, PRRSV, and IAV-S infection. A Venn diagram was applied to the expression data to elucidate the intersecting and unique genes expressed by the host in response to the viruses. (**b**) Breakdown of DEG by treatment.

The PRRSV infected samples had the largest number of statistically significant (FDR ≤ 0.1) DEGs (n = 534) followed by IAV-S (n = 184) and lastly PCV-2 (n = 119). Only the IAV-S infected samples had more upregulated genes than downregulated genes within the group. A Venn diagram (Figure 2) was applied to the expression data to elucidate the intersecting and unique genes expressed by the host in response to the viruses. The Venn diagram examines the data for processes that could differentiate active genes and pathways during singular and co-infections, focusing on the intersected sections of the diagram.

#### 2.2.1. Venn Diagram Intersection of PRRSV, PCV-2, and IAV-S

The Venn diagram (Figure 2) analysis showed that a total of 12 genes intersected across all three infections, while a total of 39 genes intersected between PRRSV and PCV-2 and a total of 50 intersected between PRRSV and IAV-S. While they differed in their expression levels (log2FC), the intersecting genes in these lists displayed the same direction of up/down expression patterns of regulation across all viruses. This allowed for the ability to investigate how divergent viruses that each affect normal respiratory functioning, but with differing pathogenicity, can affect the same gene in a similar manner. The gene lists from Figure 2 (Supplementary Table S1) were explored further to extricate candidate genes of interest intersecting between viruses involved in immune, extracellular matrix (ECM), signaling, and receptor functions. Genes of interest from the intersection of all three viruses (Table 3) included: downregulated golgin A2 (GOLGA2) a gene involved in cadherin binding and negative regulation of autophagy and peroxiredoxin 1 (PRDX1) a gene considered to be an antioxidant with anti-viral and natural-killer cell activity [27]. Upregulated genes of interest from the comparison of all three infections included: G protein signaling modulator 3 (GPSM3) involved in positive regulation of both cytokine productions related to inflammatory responses and leukocyte chemotaxis; galectin 1 (LGALS1), which is involved in the immunological processes of apoptosis, T-cell co-stimulation, as well as positive regulation of viral entry; and the RNA polymerase II subunit E (POLR2E) involved in the viral process and specifically with the influenza viral RNA transcription and replication pathways within the host [27,28,29].

#### 2.2.2. Venn Diagram Intersection between PRRSV and PCV-2

Of the 39 genes intersecting between the two infections, two-thirds were shown to be downregulated, and for every gene in the list, the expression values were larger for the PRRSV infected group. Genes of interest from the intersection of the PRRSV and PCV-2 (Table 4) included downregulated genes with both immunologic and structural integrity functions such as lymphocyte cytosolic protein 1 a gene with (LCP1) with an involvement with cell membranes, as well as being involved in T cell activation and disassembly of the extracellular matrix (ECM); amyloid beta precursor protein binding family B member 2 (APBB2) involved in apoptosis and ECM organization [27,28,29]; cadherin 5 (CDH5) involved in cell adhesion and negative regulation of inflammatory responses. There were also some genes that intersected which had strong downregulation in the PRRSV challenged animals, such as fermitin family member 2 (FERMT2) a gene involved in integrin-mediated binding and focal adhesion; syndecan 2 (SDC2), a proteoglycan involved in heparan sulfate binding and leukocyte activation; and the proteoglycan lumican (LUM), which has also been shown to function as a damage associated molecular pattern signaler (DAMP) that has been shown to be differentially expressed during other PRRSV infection studies [27,28,29,30]. Upregulated genes of interest from the PRRSV/PCV-2 intersection included RNA polymerase II subunit H (POLR2H), a member of the innate immune system and viral mRNA synthesis pathways and TP53-induced glycolysis regulatory phosphatase (TIGAR) involved in apoptosis and autophagy and can also protect host cells from reactive oxygen species (ROS) damage [31].

#### 2.2.3. Venn Diagram Intersection between PRRSV and IAV-S

The last comparison had the highest number of genes intersecting between challenges and was made from the data of intersected genes expressed during the PRRSV and IAV-S challenges. The main difference observed in this list, compared to the PRRSV/PCV-2 list, was that the majority (32/50) of intersecting DEGs were observed to be upregulated. Of interest from this list (Table 5) were the upregulated genes thioredoxin domain containing 5 (TXNDC5) involved in the negative regulation of apoptosis and neutrophil degranulation; HECT and RLD domain containing E3 ubiquitin protein ligase 5 (HERC5) involved in the biological processes of the defense response to virus, negative regulation of type I interferon production and ISG15-protein conjugation [28,32]; mitogen-activated protein kinase 14 (MAPK14) part of the MAPK signaling pathway and involved in pro-inflammatory signaling, Nucleoporin 188 (NUP188) a gene involved in antiviral ISG15 mechanisms pathways, viral processes, cellular response to stress, and is considered necessary for influenza A transcription and replication [28,33]; and C-C motif chemokine ligand 11 (CCL11), which is involved in both immunoregulatory and inflammatory biological processes, as well as monocytic, neutrophilic, and lymphocytic cell type chemotaxis activity; and ubiquitin conjugating enzyme E2 V1 (UBE2V1), a gene with multi-faceted involvement in activation of inflammatory immune responses through the formation of heterodimers. Some of the downregulated genes of interest from the PRRSV/IAV-S comparison include: fatty acid synthase (FASN), which is involved in redox and cellular responses to IL-4; atypical chemokine receptor 4 (ACKR4) involved in chemotaxis and binding of dendritic and T-cells; ATP-citrate synthase isoform 2 (ACLY), a gene involved in the innate immune system with neutrophil degranulation activity; and Pre-mRNA-processing-splicing factor 8 (PRPF8), a curious observation that functions to help with the assembly of spliceosomal proteins, as well as being involved in immune related processes such as cellular responses to lipopolysaccharide (LPS) and the tumor necrosis factor (TNF) [27,28,29,34]. 

### 2.3. Gene Set Enrichment Analysis and Pathway Analysis

In order to further investigate the contrasts and similarities among the three respiratory infections, an examination of the pathways related to intersecting genes was undertaken. Downstream analysis of the statistically significant gene lists (Supplementary Table S1) for each of the three contrasts was examined for over-enriched gene ontology (GO) terms and intersecting pathways as part of the meta-analysis of the PRRSV, PCV-2, and IAV-S sequence data. The comparison of all three pathogens only showed an intersection for a total of just 12 genes, which was not enough to test for over-enrichment, so this contrast was excluded from the analysis. Instead, more emphasis was placed on the pathways and processes in the PRRSV/PCV-2 and PRRSV/IAV-S comparisons from the Venn diagram (Figure 2).

#### 2.3.1. PRRSV and PCV-2 G.O. Analysis

**Figure 3:**
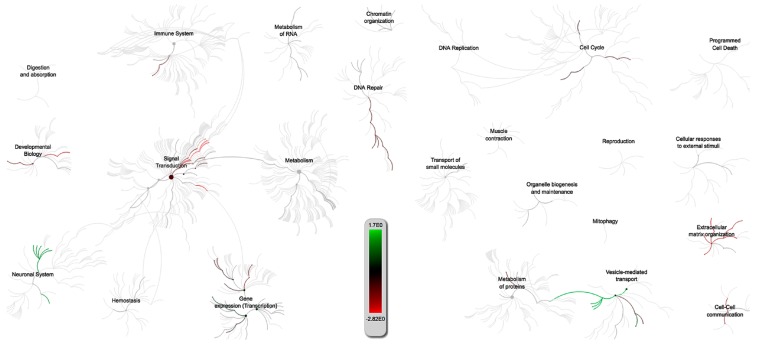
Reactome of over-represented pathways effected by gene expression changes based on the PRRSV/PCV-2 intersection from the Venn diagram (Figure 2). The scale measures the collective effect of the expressed genes in that pathway with green corresponding to upregulated and red corresponding to downregulated pathways. This is based only on *S.scrofa* pathways.

The genomic overview from reactome for the PRRSV/PCV-2 intersection showed that the DE of both infections adversely affected the immune system, DNA repair mechanisms, transcription, and cellular structural integrity pathways specific to the porcine genome (Figure 3). The genes intersecting PRRSV and PCV-2 tend towards lower expression with most interactions occurring in pathways related to signal transduction, DNA repair, and extracellular matrix organization. These results were echoed by other G.O. software that were run using both porcine and human gene annotations. Immune function pathways that appeared to be affected by both PRRSV and PCV-2 included the Fc gamma receptor (FCGR) dependent phagocytosis (R-SSC-2029480) a process of host protection in monocyte derived cells triggered by IgG for the engulfment and removal of pathogens [35]. The phagocytosis pathway was also statistically significant within the G.O results from Panther 13.1, which also showed that both viruses affect vitamin transport pathways. Another immune response related pathway observed was the TGF-beta receptor signaling pathway (GO:0007179), which has a bearing on the host immunity through regulatory action in cytokinesis and chemotaxis through interaction with the SMAD binding pathway (GO:0046332) necessary for TGF-B cellular signaling. Some of the most relevant pathway results pointed towards viral perturbation of the host cellular structural integrity that appear to also have an effect on immune signaling. The results showed statistically significant over-enrichment of the biological processes of extracellular matrix (ECM) organization (GO:0030198), non-integrin membrane-ECM interactions (R-SSC-3000171), actin filament organization (GO:0007015) and overall showed evidence of multiple pathways related to cell–cell communication such as signaling by receptor tyrosine kinases (R-SSC-9006934). Additionally, there was an intersection for the G.O. term SMAD binding (GO:0046332) involved in cellular signaling. Both the SMAD and ECM pathways mostly contained downregulated genes from the results and shared one gene in common, collagen type V alpha 2 chain (COL5A2). The overarching connection between these two respiratory infections appears to be coupled to extracellular matrix competence and signal transduction. This was observed across the different pathway results which showed suppression of multiple ECM, integrin, and cell-cell signaling pathways that may lend insight into both viral entry and host immune responses related PRRSV and PCV-2 co-infected pigs. Another significant term unique to the PRRSV/PCV-2 G.O. is the term negative regulation of mitophagy (GO:1901525), which may be a host prompted response to PRRSV infections drawing resources from the host mitochondria that work to handicap apoptotic host responses [36,37]. 

#### 2.3.2. PRRSV and IAV-S G.O. Analysis

**Figure 4:**
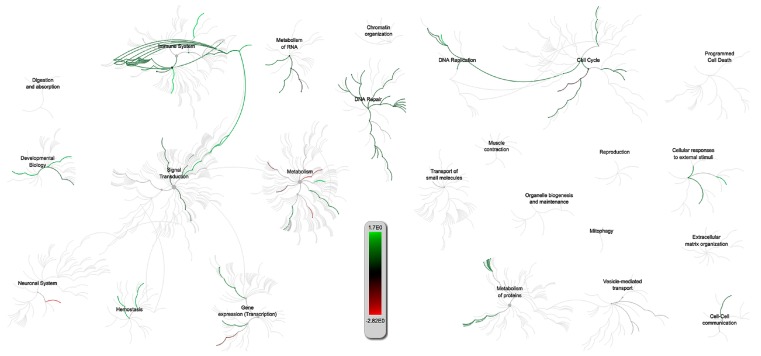
Reactome of over-represented pathways effected by gene expression changes based on the PRRSV/IAV-S intersection from the Venn diagram (Figure 2).The scale measures the collective effect of the expressed genes in that pathway with green corresponding to upregulated and red corresponding to downregulated pathways. This is based only on *S.scrofa* pathways.

The G.O. and pathway analysis for the intersection of PRRSV and IAV-S returned results that were very different than those for the PRRSV/PCV-2 intersection. Whereas, the common theme between PRRSV and PCV-2 appeared to be related to structural integrity, the PRRSV/IAV-S intersected more immune response related G.O. categories. The genome wide overview from reactome (Figure 4) indicated that the pathways intersected by PRRSV and IAV-S tend to be more upregulated with a strong connection between immune system pathways and signal transduction. Downregulation of pathways appeared to mostly affect metabolism related pathways. Many of the upregulated immune pathways fell within the innate immune pathway (R-SSC-168249) and included pathways such as neutrophil degranulation (R-SSC-6798695) in which microbiocidal granules are released, which effect the membrane structure and neutrophil activity in response to pathogens; NOD1/2 signaling pathway (R-SSC-168638) enmeshed in the pro-inflammatory response and activation of the MAPK and NF-kB pathways; and activated TAK1 mediates p38 MAPK activation (R-SSC-450302), which is involved in cytokine signaling and activation, as part of the innate immune response. Other immune related pathways with statistically significant (FDR < 0.1) G.O. hits included the oxidation-reduction process (GO:0055114), response to cytokine (GO:0034097), and response to tumor necrosis.

#### 2.3.3. Multiquery G.O. Analysis Comparison of PRRSV/PCV-2 vs. PRRSV/IAV-S

**Figure 5:**
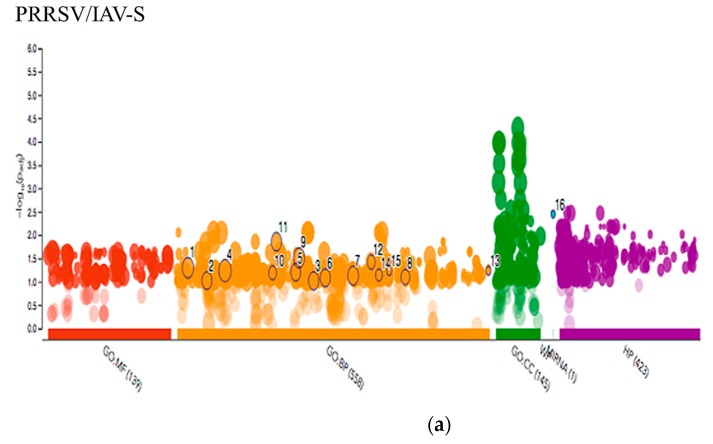

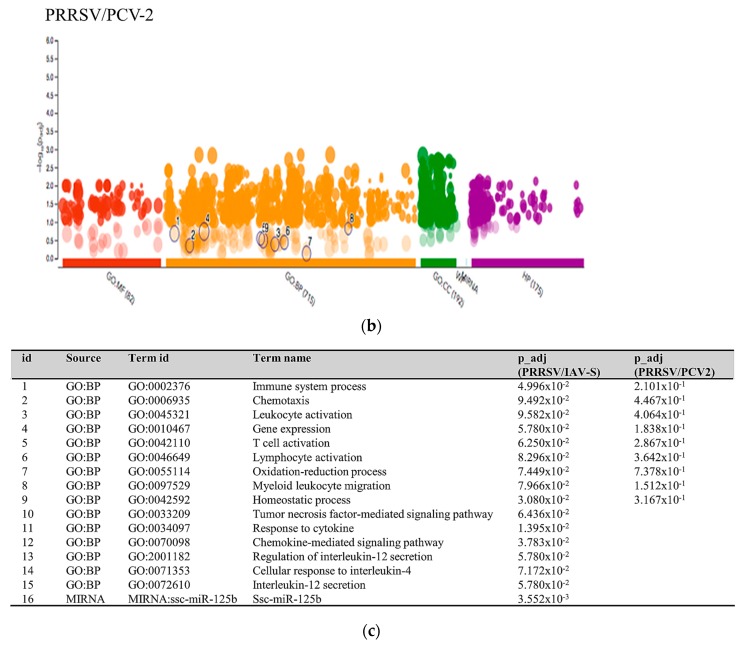
Multiquery G.O. Analysis. Manhattan plot showing G.O. enrichment. The X-axis are the G.O. functional terms colored by category. Each colored dot represents a G.O. term. The Y-axis are the adjusted –log10 p-values. Figure shows the differences in the (**a**) PRRSV/IAV-S and (**b**) PRRSV/PCV2 gene sets with focus on (**c**) terms unique or statistically significant to the PRRSV/IAV-S grouping. MF: Molecular Function; BP: Biological process; CC: Cellular component; MIRNA: MicroRNA; HP: Human Phenotype.

**Figure 6:**
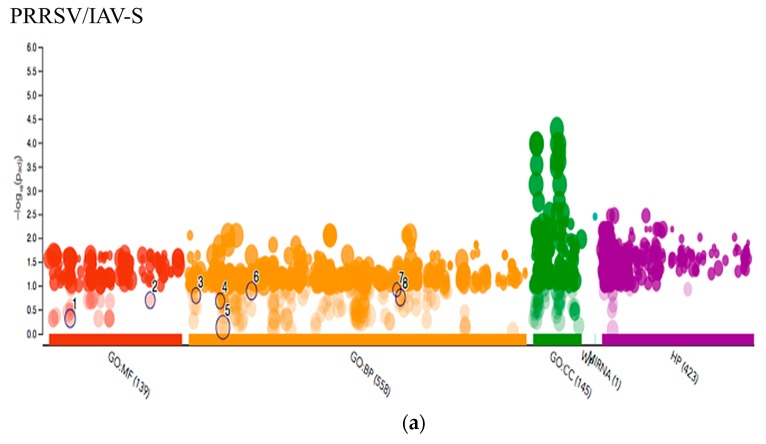

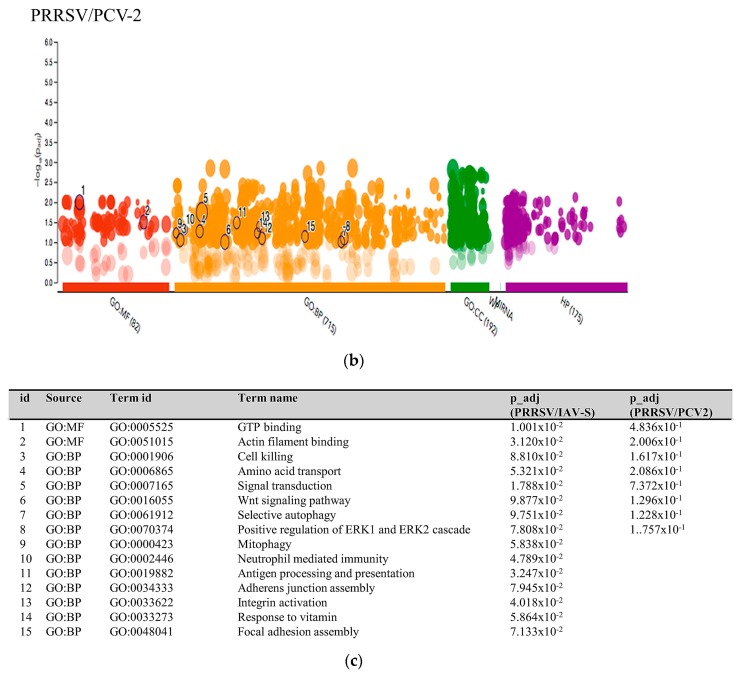
Multiquery G.O. Analysis. Manhattan plot showing G.O. enrichment. The X-axis are the G.O. functional terms colored by category. Each colored dot represents a G.O. term. The Y-axis are the adjusted –log10 p-values Figure shows the differences in the (**a**) PRRSV/IAV-S and (**b**) PRRSV/PCV2 gene sets with focus on (**c**) terms unique to or statistically significant to the PRRSV/PCV-2 grouping. MF: Molecular Function; BP: Biological process; CC: Cellular component; MIRNA: MicroRNA; HP: Human Phenotype Ontology.

The g:Profiler g:GOST functional profiling tool was also used to compare the G.O. results from each of the Venn diagram (Figure 5 and Figure 6) lists, showing an intersection with PRRSV/PCV-2 and PRRSV/IAV-S. This comparison allowed for a glimpse of what statistically significant G.O. terms were intersecting or were disparate between the two contrasts. The analysis showed that the PRRSV/IAV-grouping showed more disparate terms significant to their group involving more immune response biological procedures. This included the G.O. terms immune system process (GO:0002376), myeloid leukocyte migration (GO:0097529), chemotaxis (GO:0006935), homeostatic process (GO:0042592), T cell activation (GO:0042110) and lymphocyte activation (GO:0046649). These immune related terms were not significant within the PRRSV/PCV-2 groupings, suggesting that the terms may be more related to the IAV-S progress of infection. Additionally, the software revealed that the gene list (Supplementary Table S1) for PRRSV/IAV-S also related with ssc-miR-125b, a PRRSV anti-viral small RNA [38]. Also unique to the PRRSV/IAV-S gene intersection was the G.O. terms regulation of interleukin-12 secretion (GO:2001182), cellular response to interleukin-4 (GO:0071353), and chemokine-mediated signaling pathway (GO:0070098). However, a shift was observed within the comparison of the PRRSV/PCV-2 contrast, where less emphasis was observed on immune responses in comparison to the PRRSV/IAV-S gene intersection. The few immune related terms, however, were only statistically significant and unique to the PRRSV/PCV-2 grouping and included the Wnt signaling pathway (GO:0016055) and neutrophil mediated immunity (GO:0002446). Most of the G.O. terms in this group leaned towards binding terms and related various biological processes that may suggest that PRRSV/PCV-2 has a greater impact on organismal growth and nutrient availability. Additionally, terms such as selective autophagy (GO:0061912) and mitophagy (GO:0000423) were statistically significant and may indicate a destabilizing effect on host homeostasis that causes a synergy of illness during PRRSV/PCV-2 co-infections.

## 3. Discussion

We do not anticipate a direct effect of IAV-S on cells within lymph nodes; however, they are affected by subsequent inflammation and/or the development of pneumonia. Despite the differences in etiology, examination of these viruses allows for a comparison of the host immune response to several major porcine respiratory infections. The most notable similarities between PRRSV, IAV-S, and PCV-2 gene expression was observed in the Venn diagram (Figure 2), which showed that the genes shared between viruses experienced the same change in the regulation of expression (up or down), while only differing in magnitude. The differential in magnitude followed the known clinical course typical of experimental infection of young pigs with these viruses (i.e., PCV-2 displayed the smallest overall effect on the host gene expression, while PRRSV had the largest). Additionally supporting this was the fact that the majority of the list’s larger fold changes were observed for the PRRSV infected animals (Table 3). However, because the direction of the expression was the same across viruses, it can be said that there is a shared advantage of reducing the host ability to perform autophagy and apoptotic immune processes while concurrently promoting viral entry and disrupting NF-kB function [31]. This was most evident in the differential expression of the genes PHB2, LGALS1, POLR2E, SH3GLB1, and PRDX1. If taken collectively, the perturbation of gene expression shared across the viruses for these genes indicates very few commonalities. This could be driven mainly by the lack of intersect between PCV-2 and IAV-S seen in the Venn diagram and the difference in tropism between IAV-S and the other viruses. The most notable similarities between PRRSV, IAV-S, and PCV-2 gene expression observed in the Venn diagram intersection showed only 12 genes. The low number of intersecting genes is likely a reflection of the etiological differences between the activity of the three viruses, as well as, the difference in virulence. This difference in virulence is also reflected in the number of statistically significant genes for each virus showing evidence that the viral infections followed a clinical course in the pigs typical of experimental infection of young pigs with these viruses. The intersecting expression observed in Table 3 highlights a tendency of the three infections to modulate the host immune response through disruptions of the host’s ability to maintain homeostasis.

### 3.1. PRRSV/PCV-2 Intersection

The intersection between PRRSV and PCV-2 highlighted multiple genes involved in processes and pathways related to viral entry and replication that appear to involve adhesion and extracellular matrix interactions. Additionally, there was intersect in immune functions that involve T-cell and cytokine signaling. Of particular interest in this list was the intersection in downregulated genes with dual functions that allow them to play roles in cytokine induction and the ECM. One of these genes, LUM, is an ECM proteoglycan that also functions as a damage activated molecular pattern (DAMP) signaling gene capable of inducing inflammation [29,39,40,41]. Other proteoglycan DAMPs, such as byglycan (BGN) and decorin (DCN) have been shown to be capable of inducing inflammation and ROS production, as well as, adaptive immune signaling [29,41]. The pathway analysis along with the shared differential expression between PRRSV and PCV-2 indicates that both viruses disrupt host structural integrity and signal transduction that appears to help viral entry and replication and undermines the host ability to instigate inflammatory signaling or proper compliment activation.

### 3.2. PRRSV/IAV-S Intersection

While PRRSV and IAV-S are very different respiratory pathogens in their tropism and persistence, this comparison had the greatest number of intersecting genes related to the response to a challenge. The intersection between PRRSV and IAV-S for genes that were upregulated within the neutrophil degranulation pathway is likely related to neutrophil activity during respiratory infections. In humans and pigs this pathway has been shown to be stimulated by IAV, priming the respiratory system for the release of neutrophil granules that allow for the entry through the membrane that when maintained could damage the alveolar pathways [42,43]. For the PRRSV infected pigs, sharing in this response may possibly favor the pathogen due to the neutrophil’s ability to demolish the extra-cellular matrix during infiltration [44] and over time may contribute to the negative effects that PRRSV infection has on the integrin and focal adhesion pathways that may help in contributing to its viral infectivity. However, the upregulated genes intersecting PRRSV and IAV-S within this pathway do indicate that despite the differences in etiology there is a common response centered around neutrophilic activity that bolsters pro-inflammatory induction and oxidative responses to destroy pathogens and possibly pass on information to the adaptive immune response, through differentially expressed genes such as *RAB7A* that have antigen presenting functions [29]. It may be that, in typical IAV-S infections, this passing of information occurs, allowing for an acute sickness, whereas this does not take place with the PRRSV infection leading to a prolonged innate immune response that may contribute to the formation of lung lesions. Since the neutrophils are produced within the bone marrow, the appearance of the neutrophil degranulation pathway being perturbed within the TBLN may be related to infiltration into the lung and eventual drainage into the TBLN. Of the intersecting genes, *MAPK14* and *UBE2V1* appeared in many of the immune related G.O. pathways shared by PRRSV and IAV-S. The gene *MAPK14* is closely associated with p38 initiation which helps to stimulate IL-10 production in PRRSV infected animals. It is possible then that for PRRSV, *MAPK14* upregulation is more related to infected macrophages promoting an environment of negative regulation within the host to drive downstream IL-10 activation [45]. The upregulation of *MAPK14* in the IAV-S infected pigs, however, is likely the result of the acute nature of the virus and host induction of the pro-inflammatory cytokines to produce an antiviral environment within the respiratory tract during the early stages of infection. Another possibility is that the upregulation of *MAPK14* in the IAV-S infected pigs indicates sustained pro-inflammatory cytokine signaling that can lead to tissue damage.

## 4. Materials and Methods 

### 4.1. Ethics Statement

The animal use protocol was reviewed and approved by the Institutional Animal Care and Use Committee (IACUC) of the National Animal Disease Center-USDA-Agricultural Research Service.

### 4.2. Virus, Animals and Experimental Design

Eighty outbred weaned pigs farrowed on site out of sows from PRRSV-free commercial sources were randomly allotted to one of 4 equal treatment groups: Group 1 sham inoculated control, Group 2 PRRSV challenge, Group 3 PCV-2 challenge, or Group 4 IAV-S challenge. On 0 days post-infection (dpi) pigs received an intranasal challenge with 2 mL of either sham tissue culture supernatant or virus inoculum of a 1 × 10^5^ tissue culture infectivity dose 50% (TCID_50_) per pig according to their assigned group. Challenge viruses were PRRSV SDSU73, PCV-2 Group 2 European-like, and IAV-S H1N1 OH07, used previously in our laboratory and given at a similar dose [24,46,47]. Sham inoculum was prepared from the 3 cell cultures (MARC-145, PK-15, and MDCK cells) used to propagate the viruses. Temperatures of pigs intended for necropsy on 14 dpi were recorded daily. Pig weights were recorded on 0 dpi and at necropsy. Five pigs from each group were euthanized and necropsied on 1, 3, 6, and 14 dpi. At necropsy, lungs were scored for gross lesions. Bronchio-alveolar lavage fluid (BALF) and tracheal-bronchial lymph nodes (TBLN) were collected. Sections of TBLN and lung were placed into formalin for histopathology. A section of TBLN was homogenized and sent for flow cytometry analysis. Another section of TBLN was homogenized in tissue lysis buffer for cytokine analysis. Remaining TBLN was stored in RNA*later*™ (Thermo-Fisher scientific) at −80 °C for RNA extraction. All 0, 1, 3, 6, and 14 dpi sera, and BALF were tested for respective virus. Testing for virus included virus isolation on cell culture and/or quantitative PCR. BALF was cultured for presence of bacterial pathogens. In each treatment group, 0 and 14 dpi sera were tested for respective antibody. The in vitro assays described above are routinely performed in our laboratory [24,46,47].

### 4.3. Tissue Collection and Total RNA Isolation

One gram of TBLN from each pig was collected immediately upon necropsy, minced and stored in RNA later at −80 °C until extraction of total RNA with MagMAX™-96 for Microarrays Total RNA Isolation Kit (Applied Biosystems, Carlsbad, CA) using the manufacturer’s protocol. The integrity of the RNA was confirmed with a 2100 Bioanalyzer and RNA 6000 Nano-chip (Agilent, Santa Clara, CA, USA). The samples used had an average RIN value of 7.8 and 28S:18S rRNA ratio of 1.9. At time of collection and isolation, RNA was pooled within day for each treatment group reducing replicates.

### 4.4. Digital Gene Expression Tag Profiling (DGETP)

Tag library preparation was performed at Iowa State University DNA facility using a DGETP DpnII Sample Prep kit and protocol (Illumina, Hayward, CA, USA). In brief, total RNA aliquots (1 mg) were diluted in 50 mL of nuclease-free H2O and heated at 65 °C for 5 min to disrupt secondary structure prior to incubation with magnetic oligo-dT beads to capture the poly-adenlyated RNA fraction. First and second-strand cDNA was synthesized and bead-bound cDNA was subsequently digested with DpnII to retain a cDNA fragment from the most 3′GATC to the poly(A)-tail. Unbound cDNA fragments were washed away prior to ligation with GEX DpnII adapter to the 5′ end of the bead-bound digested cDNA fragments. This adapter contains a restriction site for MmeI which cuts 17 bp downstream from the DpnII site. After subsequent digestion with MmeI, 21 bp tags starting with the DpnII recognition sequence were recovered from the beads and dephosphorylated prior to phenol/chloroform extraction. Then, a second adapter (GEX adapter 2) was ligated onto the 3′ end of the cDNA tag at the MmeI cleavage site. The adapter-ligated cDNA tags were enriched by a 15 cycle PCR amplification using Phusion polymerase (Finnzymes) and primers complementary to the adapter sequences. The resulting fragments were purified by excision from a 6% polyacrylamide TBE gel. The DNA was eluted from the gel debris with 1× NEBuffer 2 by gentle rotation for 2 h at room temperature. Gel debris were removed using Spin-X Cellulose Acetate Filter (2 mL, 0.45 µm) and the DNA was precipitated by adding 10 µL of 3 M sodium acetate (pH 5.2) and 325 µL of ethanol (–20 °C), followed by centrifugation at 14,000 r.p.m. for 20 min. After washing the pellet with 70% ethanol, the DNA was resuspended in 10 µL of 10 mM Tris–HCl, pH8.5 and quantified with a Nanodrop 1000 spectrophotometer. Sequencing using Solexa/Illumina Whole Genome Sequencer Cluster generation was performed after applying 4 pM of each sample to the individual lanes of the Illumina 1G flowcell. After hybridization of the sequencing primer to the single-stranded products, 18 cycles of base incorporation were carried out on the 1G analyzer according to the manufacturer’s instructions. Image analysis and basecalling were performed using the Illumina Pipeline, where sequence tags were obtained after purity filtering.

### 4.5. Tag Mapping and Alignment

The raw fastq files from the SAGE sequencing run (GSE111378) were used as the input for mapping and alignment. The files were treated as single-end 3’ reads for mapping and alignment. Overall quality of the files was assessed using the FASTQC software and no trimming of reads were done due to the short SAGE read lengths. The files were aligned to the *S.scrofa* 11.1 reference genome using BWA to account for the short read length in the fastq files. Annotation of the alignment was completed using the Ensembl *S.scrofa* 11.1 gtf file and the raw read counts were calculated using the FeatureCounts software [48,49]. The default software parameters were used for all software.

### 4.6. Differential Expression Analysis

Analysis of gene expression for each of the sample groups (N = 16) was performed using the DESeq2 [50] module at usegalaxy.org [51]. Analysis was based on the main effect of treatment (Sham, PCV-2, PRRSV, and IAV-S) at 1, 3, 6, and 14 dpi. Unfortunately, due to the samples being pooled by dpi, time was not examined as an individual factor. The design formula consisted of ~treatment + time, however due to lack of biological replicates for each time point only treatment was examined. The counts were normalized using DESeq2’s Relative Log Expression (RLE) method and dispersion was estimated using the local fit type with independent filtering set to true. To elucidate the global transcriptional response during infection, comparisons between each pathogen challenged group and the sham treatment group were calculated using a statistical threshold of Q-value ≤ 0.1 to determine statistically significant differential expression (DE).

### 4.7. Gene Ontology and Pathway Analysis

Gene ontology analysis (GO) was conducted on all statistically significant differently expressed genes by the host in response to PCV-2, PRRSV, and IAV-S using a combination of multiple software and databases. Ensembl genes were converted to their gene symbol identifiers and both were used to examine over-enrichment. Genes were also converted to their human gene symbol and uniprotkb [27,29] homologs to be analyzed against both the human and pig reference genome as the background for analysis within Reactome version 65 and g:GostProfiler (version e94_eg41_p11_36d5c99) [33,52,53,54]. Statistical significance within these softwares was based on FDR ≤0.1. A comparative genomics approach was taken due to the lack of annotated gene functions available in *S.scrofa* and because it would allow for the results to be cross-checked using the software. Ensemble gene IDs that could not be converted to gene names were removed from the list prior to pathway analysis.

## 5. Conclusions

The results of this comparative study provide basic knowledge of how pigs uniquely respond to different respiratory virus infections and reveals the transcriptomic changes in the TBLN that are consistent with the relative severity of infection observed in pigs infected with these viruses.

## Figures and Tables

**Table 1:** Lung macroscopic lesion scores.

Group	3 dpi	6 dpi	14 dpi
Sham	0.34 ± 0.3	0.14 ± 0.1	0 ± 0
PCV-2	0.11 ± 0.1	1.36 ± 1.2	1.04 ± 0.6
PRRSV	5.27 ± 1.6	8.60 ± 3.4	57.1 ± 7.8
IAV-S	8.96 ± 1.4	31.5 ± 2.0	1.30 ± 0.5

Notes: PCV-2: porcine circovirus type 2; PRRSV: porcine reproductive and respiratory syndrome virus; IAV-S: influenza A virus.

**Table 2:** Virus detection by qRT-PCR.

Tissue	Treatment Group	Virus Tested for	0 dpi	1 dpi	3 dpi	6 dpi	14 dpi
Serum	Control	PCV-2	0/5	0/5	0/5	0/5	0/5
Serum	Control	PRRSV	0/5	0/5	0/5	0/5	0/5
Serum	Control	IAV-S	0/5	0/5	0/5	0/5	0/5
Serum	PCV-2	PCV-2	0/5	4/5	0/5	0/5	0/5
Serum	PRRSV	PRRSV	0/5	5/5	5/5	5/5	5/5
Serum	IAV-S	IAV-S	0/5	0/5	0/5	0/5	0/5
BALF	Control	PCV-2		0/5	0/5	0/5	0/5
BALF	Control	PRRSV		0/5	0/5	0/5	0/5
BALF	Control	IAV		0/5	0/5	0/5	0/5
BALF	PCV-2	PCV-2		4/5	5/5	4/5	4/5
BALF	PRRSV	PRRSV		3/5	5/5	5/5	5/5
BALF	IAV-S	IAV-S		0/5	1/5	1/5	0/5
TBLN	Control	PCV-2		0/5	0/5	0/5	0/5
TBLN	Control	PRRSV		0/5	0/5	0/5	0/5
TBLN	Control	IAV-S		0/5	0/5	0/5	0/5
TBLN	PCV-2	PCV-2		2/5	3/5	3/5	4/5
TBLN	PRRSV	PRRSV		5/5	5/5	5/5	5/5
TBLN	IAV-S	IAV-S		0/5	1/5	0/5	0/5

**Table 3:** List of DEGs response shared across all three viruses. All genes statistically significant at Q <= 0.1.

Gene Name	PRRSV (Log2FC)	IAV-S (Log2FC)	PCV-2 (Log2FC)	Biological Function(s)
*AK2*	1.21	0.81	0.51	Cellular energy homeostasis and hematopoiesis
*GOLGA2*	−1.51	−1.58	−1.00	Negative regulation of autophagy; cadherin binding
*GPSM3*	1.64	1.75	0.92	Positive regulation of cytokine production involved in inflammatory response, positive regulation of leukocyte chemotaxis
*LGALS1*	1.34	1.00	0.66	Apoptotic process, modulates cell-cell and cell-matrix interactions; T cell costimulation; positive regulation of viral entry into host cell; positive regulation of I-kappaB kinase/NF-kappaB signaling
*LOC100525318* (solute carrier family 23 member 1-like)	2.17	2.53	0.86	N/A
*LOC110260088* (eukaryotic translation initiation factor 3 subunit C)	2.94	2.13	1.12	N/A
*PHB2*	1.41	1.33	0.73	Negative regulation of apoptotic process, response to wounding,
*POLR2E*	1.14	0.96	0.78	Viral process
*PRDX1*	−0.85	−0.93	−0.52	Antioxidant enzyme, cadherin binding; regulation of NIK/NF-kappaB signaling; natural killer cell activation
*RBM48*	1.34	1.06	0.6	N/A
*SH3GLB1*	−0.82	−0.99	−0.63	Autophagy; regulation of cytokinesis
*SLC26A2*	0.87	1.25	0.64	Critical in cartilage for sulfation of proteoglycans and matrix organization, regulation of membrane potential

**Table 4:** List shows the intersecting genes between PRRSV and PCV-2 infections. All genes were statistically significant at Q <= 0.1.

Gene Name	PRRSV (Log2FC)	PCV-2 (Log2FC)	Biological Function(s)
*MED15*	1.90	1.03	Transcriptional coactivator in RNA pol-II transcription, cholesterol-dependent gene regulation
*POLR2H*	1.70	0.72	Encodes an essential conserved subunit of RNA poly-I, II, and III
*TMEM50B*	1.26	0.79	Late endosome to vacuole transport via multivesicular body sorting pathway
*FOLR1*	1.15	0.58	Regulation of canonical Wnt signaling, TGF-B receptor, and folate binding pathways
*STK35*	1.15	0.67	Protein phosphorylation, protein serine/threonine kinase activity
*RANBP3*	1.12	0.51	Nuclear export, negative regulator of TGF-Beta signaling
*TIGAR*	1.08	0.65	Protects cells from reactive oxygen species (ROS) and DNA damage-induced apoptosis; autophagy
*WDR1*	0.99	0.64	protein-protein interactions
*RRP9*	0.93	0.54	Component of the nucleolar small nuclear ribonucleoprotein particles (snoRNP), ribosome synthesis
*GDI1*	0.86	0.41	Regulates the GDP-GTP exchange reaction, involved cellular trafficking in organelles
*PPFIA3*	0.83	0.49	Focal adhesion disassembly
*RPL27A*	−0.59	−0.43	Structural constituent of ribosome, cytoplasmic translation
*CCDC107*	−0.87	−0.50	N/A
*RBBP4*	−0.92	−0.47	Helps form co-repressor complexes involved in transcriptional silencing, histone binding
*TMEM128*	−1.06	−0.60	Transmembrane protein
*LCP1*	−1.08	−0.55	Actin and Integrin binding; T-cell activation, Extracellular matrix disassembly
*SPG21*	−1.10	−0.62	Binds to CD4 for repression of T-cell activation, stimulatory activity of CD4, antigen receptor-mediated signaling
*RAB4A*	−1.17	−0.47	Membrane trafficking regulation, endosome sorting and recycling
*APBB2*	−1.21	−0.67	Extracellular matrix organization, positive regulation of apoptotic process
*TOPBP1*	−1.29	−0.69	N/A
*RAB11A*	−1.34	−0.70	Regulation of membrane delivery during cytokinesis, exocytosis
*NCKAP1*	−1.45	−0.52	Rac GTPase binding, apoptotic process, viral process
*NSA2*	−1.51	−0.55	Involved in cell cycle regulation and proliferation, RNA binding
*TCF12*	−1.52	−0.54	Immune response, transcriptional activator, SMAD binding
*MYOF*	−1.62	−0.74	Plasma membrane regeneration and repair, phospholipid binding, cellular response to heat
*PITHD1*	−1.75	−0.95	N/A
*CDH5 (CD144)*	−1.76	−0.73	Regulation of complement-dependent cytotoxicity, homophilic cell adhesion via plasma membrane adhesion molecules; negative regulation of inflammatory response
*CPZ*	−1.76	−0.73	Wnt signaling pathway, metallocarboxypeptidase activity
*RAB18*	−1.76	−1.15	Membrane trafficking, immune functions
*FERMT2*	−1.91	−0.68	Focal adhesion, integrin activation, cell-cell communication
*SPART*	−2.00	−0.64	Ubiquitin protein ligase binding, negative regulation of BMP signaling pathway
*COL5A2*	−2.03	−0.59	Collagen formation, SMAD binding, extracellular matrix organization
*SDC2*	−2.24	−0.77	Cell binding, cell signaling, and cytoskeletal organization, leukocyte migration
*DAAM1*	−2.38	−0.76	Rho GTPase binding
*LUM*	−2.38	−1.00	Extracellular matrix structural constituent, collagen binding, damage associated molecular pattern signaler (DAMPs)
*NTRK2*	−2.82	−1.37	MAP kinase activity, cellular response to amino acid stimulus
*GORAB*	−3.61	−1.12	Protein binding

**Table 5:** List shows the intersecting genes between PRRSV and IAV-S infections. All genes were statistically significant at Q <= 0.1.

Gene Name	PRRSV (Log2FC)	IAV-S (Log2FC)	Biological Function(s)
*ABHD17B*	−1.09	−1.09	Palmitoyl-(protein) hydrolase activity, regulation of dendritic spine maintenance
*ACKR4*	−2.19	−1.97	Receptor for C-C type chemokines; bind dendritic cell- and T cell-activated chemokines; immune response, chemotaxis
*ACLY*	−2.59	−2.46	Catalyzes the formation of acetyl-CoA and oxaloacetate, neutrophil degranulation, positive regulation of cellular metabolic process
*AIP*	2.17	2.14	Encoded protein can bind specifically to and inhibit the activity of hepatitis B virus; interleukin-12-mediated signaling pathway
*CCL11*	2.07	1.37	Antimicrobial chemokine involved in immunoregulatory and inflammatory processes; Monocyte, neutrophil, and lymphocyte chemotaxis, chronic inflammatory response
*CD5*	0.72	1.54	Type-I transmembrane glycoprotein found on the surface of thymocytes, T lymphocytes and a subset of B lymphocytes; may be involved in T cell proliferation. T cell costimulation, apoptotic signaling pathway, scavenger receptor activity
*CLRN1*	−3.08	−2.81	May be important in development and homeostasis of the inner ear and retina, actin filament organization
*CMPK1*	−0.69	−1.54	Encodes one of the enzymes required for cellular nucleic acid biosynthesis, nucleoside monophosphate kinase activity
*CTNNAL1*	−1.12	−0.98	Cell adhesion
*EIF3K*	1.45	1.24	Contributes to translation initiation factor activity, regulation of translational initiation
*EMP3*	1.00	1.04	Involved in cell proliferation, cell-cell interactions and function as a tumor suppressor, negative regulation of cell proliferation
*ETV6*	1.38	1.60	Transcription factor, negative regulation of transcription by RNA polymerase II,
*FASN*	−1.50	−1.36	Oxidation-reduction process, cellular response to interleukin-4, cadherin binding
*GAPDH*	1.35	0.99	Regulation of macroautophagy, antimicrobial humoral immune response mediated by antimicrobial peptide, positive regulation of cytokine secretion
*GINS2*	2.79	2.54	Double-strand break repair via break-induced replication, double-strand break repair via break-induced replication
*GRHPR*	2.55	2.93	Oxidoreductase activity, acting on the CH-OH group of donors, NAD or NADP as acceptor; role in metabolism
*HERC5*	2.88	2.48	Defense response to virus, negative regulation of type I interferon production, ISG15-protein conjugation, ubiquitin-protein transferase activity
*ILK*	1.08	1.25	Regulates integrin-mediated signal transduction, MAPK cascade, cell-matrix adhesion, negative regulation of cardiac muscle cell apoptotic process
*KDELC2*	1.62	1.57	Protein O-linked glycosylation via serine
*COX4I1*	0.77	0.73	Mitochondrial electron transport, cytochrome c to oxygen
*LRFN1*	−2.60	−2.15	Integral component of membrane
*LRRC8D*	0.83	1.26	Protein binding, cell volume homeostasis
*MAPK14*	2.33	2.00	MAP kinase activity, activated by various environmental stresses and proinflammatory cytokines, roles of in stress related transcription and cell cycle regulation, as well as in genotoxic stress response
*MIER1*	0.92	1.62	Transcriptional regulator
*MINDY3*	1.21	1.47	Deubiquitinase, apoptotic process, Lys48-specific deubiquitinase activity
*NCSTN*	0.89	1.23	T cell proliferation, neutrophil degranulation
*NFIX*	1.30	1.24	Transcriptional activator activity, RNA polymerase II transcription regulatory region sequence-specific DNA binding
*NUP188*	1.50	1.65	Viral process, structural constituent of nuclear pore
*OIP5*	1.12	1.56	CENP-A containing nucleosome assembly, protein binding
*PLA2G2D*	1.40	1.19	Gene may be involved in inflammation and immune response, inflammatory response, negative regulation of T cell proliferation, heparan sulfate proteoglycan binding
*PRPF3*	1.01	0.83	Pre-mRNA processing factor, mRNA splicing, via spliceosome
*PRPF8*	−3.25	−2.43	Cellular response to lipopolysaccharide, cellular response to tumor necrosis factor, mRNA splicing, via spliceosome
*PTDSS1*	1.52	1.57	Transferase activity, phosphatidylserine biosynthetic process
*RAB7A*	1.07	1.26	Phagosome-lysosome fusion, neutrophil degranulation, antigen processing and presentation of exogenous peptide antigen via MHC class II (ALL HUMAN)
*RBM26*	−1.37	−1.22	Regulation of mRNA processing
*RFC2*	1.11	1.32	Binding ATP and may help promote cell survival; DNA damage response, detection of DNA damage
*RPF1*	−0.71	−1.15	rRNA processing
*SMARCA5*	−1.16	−1.67	Regulation of transcription by RNA polymerase II, cellular response to leukemia inhibitory factor
*SNX8*	2.25	2.90	Intracellular protein transport, phosphatidylinositol binding
*SORBS2*	−3.49	−3.29	Actin filament organization, cytoskeletal adaptor activity
*SZRD1*	1.42	1.15	N/A
*TSEN54*	1.08	1.00	Removal of introns from precursor tRNAs, tRNA splicing, via endonucleolytic cleavage and ligation
*TXNDC5*	3.30	1.75	Role may be to protect hypoxic cells from apoptosis, cell redox homeostasis, negative regulation of apoptotic process, neutrophil degranulation
*UBE2V1*	1.29	1.12	Positive regulation of NF-kappaB transcription factor activity, activation of MAPK activity, positive regulation of I-kappaB kinase/NF-kappaB signaling
*UTRN*	−2.66	−2.57	Positive regulation of cell-matrix adhesion, integrin binding
*WDFY2*	1.13	0.82	Positive regulation of fat cell differentiation, positive regulation of protein phosphorylation

## Supplementary Materials

The following are available online at https://www.mdpi.com/2076-0817/9/2/99/s1, Table S1: Gene lists from differential gene expression analysis by virus. Columns show GeneID, log2(FC), and P-adj data for each treatment group. These gene list were used as the query lists for network prediction. The fold changes reported are the difference in expression values for each gene.

## References

[1] Holtkamp D.J., Kliebenstein J.B., Neumann E., Zimmerman J.J., Rotto H., Yoder T.K., Wang C., Yesk P.E., Mowrer C.L., Haley C.A. (2013). Assessment of the economic impact of porcine reproductive and respiratory syndrome virus on United States pork producers. J. Swine Health Product..

[2] Nieuwenhuis N., Duinhof T.F., Van Nes A. (2012). Economic analysis of outbreaks of porcine reproductive and respiratory syndrome virus in nine sow herds. Veter. Rec..

[3] Simionatto S., Marchioro S.B., Maes M., Dellagostin O.A. (2013). Mycoplasma hyopneumoniae: From disease to vaccine development. Veter. Microbiol..

[4] Dobrescu I., Levast B., Lai K., Delgado-Ortega M., Walker S., Banman S., Townsend H., Simon G., Zhou Y., Gerdts V. (2014). In vitro and ex vivo analyses of co-infections with swine influenza and porcine reproductive and respiratory syndrome viruses. Veter. Microbiol..

[5] Gale M., Tan S.-L., Katze M.G. (2000). Translational Control of Viral Gene Expression in Eukaryotes. Microbiol. Mol. Boil. Rev..

[6] Walsh D., Mohr I. (2011). Viral subversion of the host protein synthesis machinery. Nat. Rev. Genet..

[7] Miller S., Krijnse-Locker J. (2008). Modification of intracellular membrane structures for virus replication. Nat. Rev. Genet..

[8] Guidotti L.G., Chisari F.V. (2001). Noncytolytic Control Of Viral Infections By The Innate And Adaptive Immune Response. Annu. Rev. Immunol..

[9] Barra N.G., Gillgrass A., Ashkar A. (2010). Effective control of viral infections by the adaptive immune system requires assistance from innate immunity. Expert Rev. Vaccines.

[10] Glass E.J., Baxter R., Leach R.J., Jann O.C. (2012). Genes controlling vaccine responses and disease resistance to respiratory viral pathogens in cattle. Vet. Immunol. Immunopathol..

[11] Iwasaki A., Medzhitov R. (2015). Control of adaptive immunity by the innate immune system. Nat. Immunol..

[12] Miller L.C., Bayles D.O., Zanella E.L., Lager K.M. (2015). Effects of Pseudorabies Virus Infection on the Tracheobronchial Lymph Node Transcriptome. Bioinform. Biol. Insights..

[13] Miller L.C., Fleming D., Arbogast A., O Bayles D., Guo B., Lager K.M., Henningson J.N., Schlink S.N., Yang H.-C., Faaberg K.S. (2012). Analysis of the swine tracheobronchial lymph node transcriptomic response to infection with a Chinese highly pathogenic strain of porcine reproductive and respiratory syndrome virus. BMC Veter- Res..

[14] Meng X.-J. (2012). Spread like a wildfire—The omnipresence of porcine circovirus type 2 (PCV2) and its ever-expanding association with diseases in pigs. Virus Res..

[15] Rajao D.S., Vincent A.L. (2015). Swine as a Model for Influenza A Virus Infection and Immunity. ILAR J..

[16] Talker S.C., Stadler M., Koinig H.C., Mair K.H., Rodríguez-Gómez I.M., Graage R., Zell R., Dürrwald R., Starick E., Harder T. (2016). Influenza A Virus Infection in Pigs Attracts Multifunctional and Cross-Reactive T Cells to the Lung. J. Virol..

[17] Chang H.-W., Jeng C.-R., Lin T.-L., Liu J.J., Chiou M.-T., Tsai Y.-C., Chia M.-Y., Jan T.-R., Pang V.F. (2006). Immunopathological effects of porcine circovirus type 2 (PCV2) on swine alveolar macrophages by in vitro inoculation. Veter. Immunol. Immunopathol..

[18] Segalés J. (2012). Porcine circovirus type 2 (PCV2) infections: Clinical signs, pathology and laboratory diagnosis. Virus Res..

[19] Borghetti P., Morganti M., Saleri R., Ferrari L., De Angelis E., Cavalli V., Cacchioli A., Corradi A., Martelli P. (2013). Innate pro-inflammatory and adaptive immune cytokines in PBMC of vaccinated and unvaccinated pigs naturally exposed to porcine circovirus type 2 (PCV2) infection vary with the occurrence of the disease and the viral burden. Veter. Microbiol..

[20] Harbers M., Carninci P. (2005). Tag-based approaches for transcriptome research and genome annotation. Nat. Methods.

[21] Morrissy S., Zhao Y., Delaney A., Asano J., Dhalla N., Li I., McDonald H., Pandoh P., Prabhu A.-L., Tam A. (2010). Digital Gene Expression by Tag Sequencing on the Illumina Genome Analyzer. Curr. Protoc. Hum. Genet..

[22] De Lorgeril J., Zenagui R., Rosa R.D., Piquemal D., Bachère E. (2011). Whole Transcriptome Profiling of Successful Immune Response to Vibrio Infections in the Oyster Crassostrea gigas by Digital Gene Expression Analysis. PLoS ONE.

[23] Jin W., Olson E.N., Moore S.S., Basarab J.A., Basu U., Guan L.L. (2012). Transcriptome analysis of subcutaneous adipose tissues in beef cattle using 3′ digital gene expression-tag profiling1. J. Anim. Sci..

[24] Lager K.M., Gauger P.C., Vincent A.L., Opriessnig T., Kehrli M.E., Cheung A.K. (2007). Mortality in pigs given porcine circovirus type 2 subgroup 1 and 2 viruses derived from DNA clones. Veter. Rec..

[25] Mengeling W.L., Lager K.M., Vorwald A.C. (1998). Clinical consequences of exposing pregnant gilts to strains of porcine reproductive and respiratory syndrome (PRRS) virus isolated from field cases of “atypical” PRRS. Am. J Vet. Res..

[26] Vincent A.L., Ma W., Lager K.M., Gramer M.R., Richt J.A., Janke B.H. (2009). Characterization of a newly emerged genetic cluster of H1N1 and H1N2 swine influenza virus in the United States. Virus Genes.

[27] UniProt C (2015). UniProt: A hub for protein information. Nucleic Acids Res..

[28] Jenuth J.P. (2000). The NCBI. Publicly available tools and resources on the Web. Methods Mol. Boil. (Clifton, N.J.).

[29] The UniProt C (2017). UniProt: The universal protein knowledgebase. Nucleic Acids Res..

[30] Miller L.C., Fleming D.S., Li X., Bayles D.O., Blecha F., Sang Y. (2017). Comparative analysis of signature genes in PRRSV-infected porcine monocyte-derived cells to different stimuli. PLoS ONE.

[31] Chen Q., Kang J., Fu C. (2018). The independence of and associations among apoptosis, autophagy, and necrosis. Signal Transduct. Target. Ther..

[32] Wong J.J.Y., Pung Y.F., Sze N.S.-K., Chin K.-C. (2006). HERC5 is an IFN-induced HECT-type E3 protein ligase that mediates type I IFN-induced ISGylation of protein targets. Proc. Natl. Acad. Sci. USA.

[33] Fabregat A., Jupe S., Matthews L., Sidiropoulos K., Gillespie M., Garapati P., Haw R., Jassal B., Korninger F., May B. (2018). The Reactome Pathway Knowledgebase. Nucleic Acids Res..

[34] Brown G.R., Hem V., Katz K.S., Ovetsky M., Wallin C., Ermolaeva O., Tolstoy I., Tatusova T., Pruitt K.D., Maglott D.R. (2015). Gene: A gene-centered information resource at NCBI. Nucleic Acids Res..

[35] Joshi T., Butchar J., Tridandapani S. (2006). Fcγ Receptor Signaling in Phagocytes. Int. J. Hematol..

[36] Zhang L., Qin Y., Chen M. (2018). Viral strategies for triggering and manipulating mitophagy. Autophagy.

[37] Li S., Wang J., Zhou A., Khan F.A., Hu L., Zhang S. (2016). Porcine reproductive and respiratory syndrome virus triggers mitochondrial fission and mitophagy to attenuate apoptosis. Oncotarget.

[38] Li J., Chen Z., Zhao J., Fang L., Fang R., Xiao J., Chen X., Zhou A., Zhang Y., Ren L. (2015). Difference in microRNA expression and editing profile of lung tissues from different pig breeds related to immune responses to HP-PRRSV. Sci. Rep..

[39] Carbon S., Ireland A., Mungall C.J., Shu S., Marshall B., Lewis S., AmiGO H., Web Presence Working Group (2009). AmiGO: Online access to ontology and annotation data. Bioinform..

[40] Moreth K., Iozzo R.V., Schaefer L. (2012). Small leucine-rich proteoglycans orchestrate receptor crosstalk during inflammation. Cell Cycle.

[41] Nikitovic D., Papoutsidakis A., Karamanos N.K., Tzanakakis G.N. (2014). Lumican affects tumor cell functions, tumor–ECM interactions, angiogenesis and inflammatory response. Matrix Boil..

[42] Kennedy J.L., Turner R.B., Braciale T., Heymann P.W., Borish L. (2012). Pathogenesis of rhinovirus infection. Curr. Opin. Virol..

[43] Borregaard N., Sørensen O.E., Theilgaard-Mönch K. (2007). Neutrophil granules: A library of innate immunity proteins. Trends Immunol..

[44] Camp J.V., Jonsson C.B. (2017). A Role for Neutrophils in Viral Respiratory Disease. Front. Immunol..

[45] Hou J., Wang L., Quan R., Fu Y., Zhang H., Feng W.-H. (2012). Induction of interleukin-10 is dependent on p38 mitogen-activated protein kinase pathway in macrophages infected with porcine reproductive and respiratory syndrome virus. Virol. J..

[46] Lager K.M., Halbur P.G. (1996). Gross and Microscopic Lesions in Porcine Fetuses Infected with Porcine Reproductive and Respiratory Syndrome Virus. J. Veter- Diagn. Investig..

[47] Vincent A.L., Ma W., Lager K.M., Janke B.H., Webby R.J., García-Sastre A., Richt J.A. (2007). Efficacy of intranasal administration of a truncated NS1 modified live influenza virus vaccine in swine. Vaccine.

[48] Brown J., Pirrung M., McCue L.A. (2017). FQC Dashboard: Integrates FastQC results into a web-based, interactive, and extensible FASTQ quality control tool. Bioinform..

[49] Liao Y., Smyth G.K., Shi W. (2014). Feature Counts: An efficient general purpose program for assigning sequence reads to genomic features. Bioinform..

[50] I Love M., Huber W., Anders S. (2014). Moderated estimation of fold change and dispersion for RNA-seq data with DESeq2. Genome Boil.

[51] Afgan E., Baker D., Beek M.V.D., Blankenberg D., Bouvier D., Čech M., Chilton J., Clements D., Coraor N., Eberhard C. (2016). The Galaxy platform for accessible, reproducible and collaborative biomedical analyses: 2016 update. Nucleic Acids Res..

[52] Fabregat A., Sidiropoulos K., Viteri G., Forner O., Marin-Garcia P., Arnau V., D’Eustachio P., Stein L., Hermjakob H. (2017). Reactome pathway analysis: A high-performance in-memory approach. BMC Bioinform..

[53] Reimand J., Arak T., Adler P., Kolberg L., Reisberg S., Peterson H., Vilo J. (2016). g:Profiler-a web server for functional interpretation of gene lists (2016 update). Nucleic Acids Res..

[54] Reimand J., Kull M., Peterson H., Hansen J., Vilo J. (2007). g:Profiler—A web-based toolset for functional profiling of gene lists from large-scale experiments. Nucleic Acids Res..

